# A comparison of the microbiology profile for periprosthetic joint infection of knee arthroplasty and lower-limb endoprostheses in tumour surgery

**DOI:** 10.5194/jbji-7-177-2022

**Published:** 2022-08-08

**Authors:** Robert A. McCulloch, Amirul Adlan, Neil Jenkins, Michael Parry, Jonathan D. Stevenson, Lee Jeys

**Affiliations:** 1 Bone Infection Service, The Royal Orthopaedic Hospital, Birmingham, UK; 2 Department of Infectious Diseases and Tropical Medicine, University Hospitals Birmingham, Birmingham, UK; 3 School of Life and Health Sciences, Aston University, Birmingham, UK

## Abstract

**Aims**: this study compared the patient and microbiological profile of prosthetic joint infection (PJI) for patients treated with two-stage revision for knee arthroplasty with that of lower-limb endoprostheses for oncological resection. **Patient and methods**: a total of 118 patients were treated with two-stage revision surgery for infected knee arthroplasty and lower-limb endoprostheses between 1999 and 2019. A total of 74 patients had two-stage revision for PJI of knee arthroplasty, and 44 had two-stage revision of oncology knee endoprostheses. There were 68 men and 50 women. The mean ages of the arthroplasty and oncology cohorts were 70.2 years (range of 50–89) and 36.1 years (range of 12–78) respectively ( p<0 .01). Patient host and extremity criteria were categorized according to the Musculoskeletal Infection Society (MSIS) host and extremity staging system. The patient microbiological culture, the incidence of polymicrobial infection, and multidrug resistance (MDR) were analysed and recorded. **Results**: polymicrobial infection was reported in 16 % (12 patients) of knee arthroplasty PJI cases and in 14.5 % (8 patients) of endoprostheses PJI cases ( p=0 .783). There was a significantly higher incidence of MDR in endoprostheses PJI, isolated in 36.4 % of cultures, compared with knee arthroplasty PJI (17.2 %, p=0 .01). Gram-positive organisms were isolated in more than 80 % of cultures from both cohorts. Coagulase-negative *Staphylococcus* (CoNS) was the most common Gram-positive organism, and *Escherichia coli* was the most common Gram-negative organism in both groups. According to the MSIS staging system, the host and extremity grades of the oncology PJI cohort were significantly worse than those for the arthroplasty PJI cohort ( p<0 .05). **Conclusion**: empirical antibiotic prophylaxis against PJI in orthopaedic oncology is based upon PJI in arthroplasty, despite oncology patients presenting with worse host and extremity staging. CoNS was the most common infective organism in both groups; however, pathogens showing MDR were significantly more prevalent in oncological PJI of the knee. Therefore, empirical broad-spectrum treatment is recommended in oncological patients following revision surgery.

## 1 Introduction

The majority of patients presenting with bone and soft-tissue sarcomas are managed with limb-salvage surgery (Cirstoiu et al., 2019; Wafa and Grimer, 2006). Limb-salvage surgery with endoprosthetic reconstruction (EPR) provides a good level of function with an acceptable implant survivorship of over 80 % at 10 years (Gosheger et al., 2006). However, the complication profile compared with that of primary arthroplasty is significantly higher. The prosthetic joint infection (PJI) rate of proximal and distal femoral EPR for sarcoma is approximately 10 % compared with approximately 1 % in primary hip and knee arthroplasty (Racano et al., 2013; Kapadia et al., 2016). Pulido et al. (2008) described multiple independent predictors for PJI including patient morbidity, allogenic transfusion, and longer hospitalization. Orthopaedic oncology patients are at a higher risk of PJI due to their immunosuppression secondary to neo-adjuvant chemoradiotherapy and having an underlying malignancy, longer and more extensive operative procedures, higher transfusion rates due to levels of soft-tissue dissection, and increased length of stay than primary arthroplasty patients.

Although successful eradication of PJI in primary hip and knee arthroplasty may be quoted to be as high as 90 %, whether using a single-stage or two-stage protocol, these results are not reproduced within the context of EPRs due to multifactorial local and systemic disparities (Kapadia et al., 2016).

Perioperative antibiotic prophylaxis for EPRs is typically based upon local guidance for non-oncological arthroplasty to cover common infective organisms (Christensen et al., 2021). In oncological PJI, the most common infective organism is reportedly coagulase-negative *Staphylococcus*, mirroring that of primary arthroplasty PJI (Jeys et al., 2005).

The aim of this study was to compare the microbiological organisms responsible for PJI in patients who underwent two-stage revision of infected primary total knee arthroplasty (TKA) with those of patients who underwent two-stage revision of infected oncological EPRs of the knee at a single institution. Current antibiotic prophylaxis for oncological patients is based upon evidence from primary arthroplasty, despite significant differences in both patient and procedure. This will subsequently guide decision-making regarding antibiotic prophylaxis at primary implantation for oncological procedures and empirical antibiotics for infected revision procedures (where the infecting organism(s) are unknown).

## 2 Patients and methods

After local approval, a retrospective analysis of the departmental PJI database was conducted to identify a consecutive cohort of patients who underwent two-stage revision surgery for infected primary TKA and patients with infected EPRs of the lower-limb following tumour resection at a tertiary arthroplasty and oncology centre in the United Kingdom between 1999 and 2019.

Inclusion in the study was defined as confirmed PJI defined using the Musculoskeletal Infection Society International Consensus Meeting (MSIS ICM; Parvizi et al., 2018). As a comparator group to oncological patients managed with staged revision for PJI, a consecutive cohort of staged revision for PJI of a primary TKA were identified. Two-stage revision is the current standard of practice for the surgical management of infected TKA within our institution. Patients were excluded from both cohorts if any previous procedures to manage their infection prior to their first-stage procedure (e.g. washout or debridement and implant retention procedure) had taken place. A minimum of 2 years of follow-up was required for inclusion. Antibiotic prophylaxis for all primary procedures in both groups was flucloxacillin and gentamicin within 30 min of knife to skin with three post operative doses of flucloxacillin. If the patient was allergic to penicillin, teicoplanin was used as an alternative to flucloxacillin. During the first-stage revision, vancomycin and meropenem were used empirically with second-stage antibiotic prophylaxis; this was decided upon during the preoperative bone infection multidisciplinary team (MDT) discussion.

Polymicrobial infection was defined as the presence of two or more infecting organisms. Multidrug resistance (MDR) was defined as the presence of a single organism resistant to three or more antimicrobial classes (Parvizi et al., 2018). All patients were classified according to the MSIS host and extremity staging system which categorizes host status, including comorbidity, and soft-tissue status in lower-limb PJI patients (Fehring et al., 2017). A systematic sampling method was undertaken in the theatre to minimize the risks of contamination. A minimum of five samples were sent for microbiological analysis at each first- and second-stage procedure, and antibiotics were withheld prior to surgery unless the patient was systemically septic and compromised. All cases were Gram stained and cultured by direct and enrichment methods for 15 d along with antibiotic susceptibility testing. Only organisms that were cultured from at least two samples were included in this dataset.

## 3 Statistical analysis

Statistical analysis was performed with IBM SPSS Statistics 24.0 (IBM, Armonk, New York, USA). Median and mean values with ranges were calculated for continuous variables. A chi-square test was used to test statistical significance for categorical variables. An independent t test was conducted for normally distributed continuous variables. A Mann–Whitney U test was conducted for non-normally distributed variables. A p value of < 0.05 was set to be statistically significant.

## 4 Results

A total of 118 continuous patients were identified as having undergone two-stage revision surgery to eradicate PJI between 1999 and 2019 in both groups: 74 patients had PJI following primary TKA, and 44 patients had PJI following oncological resection and reconstruction with a distal femoral EPR. All microbiological data are available in Table S1 in the Supplement. There was no significant difference in sex between the two groups ( p=0 .729). The mean ages for the TKA cohort and lower-limb EPR cohort were 70.2 years (range of 50–89) and 36.1 years (range of 12–78) respectively ( p<0 .01). The patients and limb status for both groups were categorized according to the MSIS staging system for host and extremity, as shown in Table 1. Patients with infected lower-limb EPRs were noted to be significantly worse hosts, with the majority of patients categorized in grade C (66 %), whereas most patients with PJI following TKA were grade A (39 %) or B (45 %) ( p<0 .001, chi-square test). Regarding extremity status, the TKA group had a better limb status (63 % in grade 1 and 21 % in grade 2) compared with the group with infected EPR (95 % of patients in grade 2) (chi-square test, p<0 .001). The mean time to first-stage revision for the TKA group was 67.5 months (range of 2–267). Six patients had a first-stage revision within 6 months of the primary procedure. The mean time to first-stage revision for the infected EPR group was 96.1 months (range of 2–397) with two patients having their first-stage revision within 6 months.

**Figure 1:**
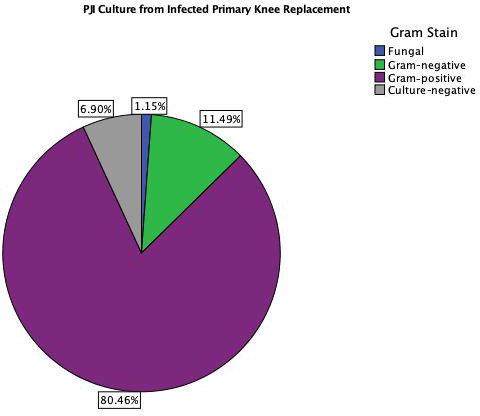
PJI culture results from infected primary TKA.

**Figure 2:**
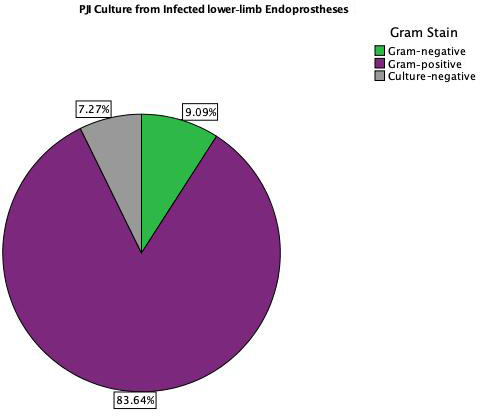
PJI culture results from infected lower-limb EPR.

The distributions of isolated organisms based on Gram staining for both groups (primary TKA PJI vs. EPR PJI) are shown in Figs. 1 and 2 respectively. More than 80 % of isolated organisms were Gram-positive in both groups. The most common Gram-positive organism for both groups was coagulase-negative *Staphylococcus* (CoNS), which was isolated in 51 % of the Gram-positive organisms from primary TKA PJI and in 50 % of the Gram-positive organisms from the endoprosthetic PJI. The most common Gram-negative organism was *Escherichia coli* in both groups, which was isolated in 30 % (3 of 10) of the Gram-negative organisms from the primary TKA PJI and in 40 % (2 of 5) of the Gram-negative organisms from the EPR PJI group.

**Table 1:** Patient and limb status for both groups.

	Primary TKA PJI	Lower-Limb EPR PJI	p value
N	74	44	
Men (% of group)	43 (58.1)	25 (56.8)	0.73
Women (% of group)	31 (41.9)	19 (43.2)	
Age (% of group)	70.2 (50–89)	36.1 (12–78)	2.92
MSIS host, N (%)			
A	34 (39.1)	5 (9.1)	≤<0.01
B	39 (44.8)	3 (5.5)	1.41
C	1 (1.1)	36 (65.5)	2.24
MSIS limb/extremity, N (%)			
1	55 (63.2)	0	1.69
2	18 (20.7)	42 (95.4)	3.18
3	1 (1.1)	2 (4.5)	0.55
MSIS host and extremity, N (%)			
A1	25 (33.8)	0	≤0.01
A2	9 (12.2)	5 (11.4)	1.00
B1	29 (39.2)	0	9.02
B2	9 (12.2)	2 (4.5)	0.21
B3	0	1 (2.3)	0.37
C1	1 (1.4)	0	1.00
C2	0	35 (79.5)	8.77
C3	0	1 (2.3)	0.38
Polymicrobial, N (%)	12 (16.2)	8 (14.5)	0.78
Multidrug resistance (MDR), N (%)	15 (17.2)	20 (36.4)	0.01

Multiple comparisons with Bonferroni correction were conducted for all organisms by species. There were no significant differences amongst the infecting organisms between the TKA and EPR patients ( p>0 .05). The incidence of PJI with CoNS as the causative organism was reported to have an increasing trend from 2000 up to 2015 followed by a decline towards the end of the study period. Similarly, the incidence of PJI due to *Staphylococcus aureus* also peaked in 2015.

There was no difference in the incidence of polymicrobial infection in both groups, with an incidence of 16 % in the infected TKA group and an incidence of 15 % in the infected EPR group ( p=0 .783, chi-square test). However, the incidence of multidrug resistant organisms (MDROs) in EPR PJI was 36 %, which was significantly higher than the primary TKA group with an incidence of 17 % ( p=0 .01, chi-square test). The highest incidence of MDR was observed in coagulase-negative *Staphylococcus* (80 % of all MDROs) followed by *Enterococcus* spp. (6 %) and other organisms, including *Klebsiella* spp., *Kocuria* spp., *Serratia* spp., and vancomycin-resistant *Enterococci* (VRE). Comparing the infected primary arthroplasty and the infected endoprosthesis group, there was no statistically significant difference regarding the organisms grown, with both groups reporting CoNS as the most common MDRO grown ( p=0 .548). A total of 10 patients (22.7 %) in the oncological group had previously had radiotherapy, and 34 patients (77.3 %) had undergone chemotherapy. Six oncology patients had local flap coverage at the time of their index surgery. A total of 31 patients (70.5 %) had silver-coated implants for their second-stage revision surgery. Within the oncological group, there were no statistically significant differences in MDR rates depending upon whether the patient had undergone radiotherapy ( p=0 .15) or chemotherapy ( p=0 .62) or whether they had flap coverage ( p=0 .48) or silver-coated implants ( p=0 .44).

In the oncological patient group with infected EPR, 6 patients (13.6 %) demonstrated resistance to gentamicin, and 16 patients (36.4 %) showed resistance to flucloxacillin. In the primary arthroplasty group with infected EPRs, 11 patients (12.6 %) showed resistance to gentamicin, and 15 patients (17.2 %) showed resistance to flucloxacillin. Comparing the two cohorts, patients with an infected EPR demonstrated a higher proportion of resistance to flucloxacillin than infected primary arthroplasty patients, but there was no statistical significance achieved. (36.4 % vs. 17.2 %; p=0 .098, chi-square test). Regarding MDR cases, in the infected EPR group, 77.8 % (14) of cases showed resistance to flucloxacillin, whereas 27.8 % (5) of cases showed resistance to gentamicin. In the primary arthroplasty cohort with MDROs, 13.5 % (10) of cases showed resistance to flucloxacillin, and 10.8 % (8) of cases showed resistance to gentamicin.

## 5 Discussion

Our study demonstrated a significantly higher incidence of MDROs in oncological PJI compared with TKA PJI (36 % vs. 17 %; p=0 .01). In 80 % of MDR cases (28 of 35), the organism was CoNS. VRE and methicillin-resistant *Staphylococcus aureus* (MRSA) were isolated as causative agents of PJI in infected EPRs, whereas none were reported in the primary arthroplasty group.

PJI remains one of the most devastating complications following primary joint arthroplasty and endoprosthetic reconstruction surgery. The pathogenesis of PJI is either by intraoperative inoculation, haematogenous spread after implantation, or direct contact with nearby infected tissues (Li et al., 2018). Prevention of acute PJI is multifactorial, including patient optimization and intra-operative factors such as skin preparation, draping, and prophylactic antibiotics. It has been known for over 50 years that prophylactic antibiotics are one of the most potent measures for preventing PJI (Fogelberg et al., 1970). Appreciation of a local and up-to-date antibiogram guides mibrobiologists in deciding upon the preferred antibiotic prophylaxis (Bosco et al., 2015).

Although PJI can begin with an MDRO, such as *Acinetobacter* spp., it is more common that antibiotic drug resistance is acquired through mechanisms such as prevention of access to drug target (e.g. reduced membrane permeability), alteration of drug target (mutational or non-mutational), or drug disruption (e.g. hydrolytic degradation) (Zmistowski and Alijanipour, 2014). Previously, Dhanoa et al. (2015) reported MDR in 52.6 % of the isolated strains in orthopaedic oncology patients. The higher incidence of MDR in the orthopaedic oncological group in our study may be attributed to the poor host and extremity criteria, with the majority of patients categorized as host grade C and limb status grade 2 or 3 according to the MSIS staging system (Fehring et al., 2017). This may be explained by their exposure to compromising factors, such as previous chemotherapy or radiotherapy, that increased their risks of recurrent antimicrobial therapy for hospital- or community-acquired infections. Initial undertreatment of PJI with antibiotics can also increase the risk of drug resistance (Li et al., 2020). Specifically related to coagulase-negative *Staphylococcus* species, selection pressures on commensal bacteria from antibiotics in healthcare settings and possible cross-contamination of resistant organisms between patients and healthcare staff can result in preoperative skin colonization or post-operative wound colonization by multidrug resistant species (Pantosti et al., 2007).

The incidence of polymicrobial infection in our study was 16.2 % in primary TKA PJI cases and 14.5 % in EPR PJI cases. Risk factors for polymicrobial PJI include host factors such as obesity, diabetes, and peripheral vascular disease (McPherson et al., 2002). The previous literature has reported a polymicrobial incidence of 4 %–27 % of all PJI (Gallo et al., 2006; Tsukayama et al., 1996) compared with a multi-institutional study by Morii et al. (2013) assessing the outcomes of deep infection in oncology EPRs of the knee with a polymicrobial incidence rate of 3.5 %. Within the oncology group, the mean age was lower; thus, associated age-related comorbid factors were largely absent. Despite this, the polymicrobial rate in the oncology group was equivalent to that of the arthroplasty group. This suggests that the oncological patients are independently at risk of polymicrobial infection.

CoNS was the most common Gram-positive organism responsible for PJI in both groups, accounting for 41 % of total organisms isolated in PJI of primary TKA surgery and 42 % in PJI of the oncology EPR in our study. This is consistent with previous oncology and arthroplasty literature (Nickinson et al., 2010; Jeys and Grimer, 2009). The most common Gram-negative organisms were *Escherichia coli* in 3.4 % and 3.6 % of the knee arthroplasty PJI and oncological PJI respectively, which is also consistent with previous reports describing the epidemiology of PJI (Tsai et al., 2019; Legout et al., 2006).

There are several limitations to the present study, including its retrospective data collection which may lead to reporting biases. Revision of primary TKA cases for PJI was chosen as a comparator group to the oncological PJI group because perioperative antibiotic protocols for PJI are typically based upon this group and the typical microbiology from these cases. However, there were differences between groups with respect to age and MSIS grading; therefore, although these are close comparator groups, biases may stem from the differences in age-related comorbid/host status and soft-tissue status. Due to the application of the ICM (Parvizi et al., 2018) classification of PJI to our dataset from 1999 to 2019, not all novel diagnostic methods were available during the study period (e.g. synovial markers such as α -defensin and D-dimer). Therefore, during this time, patients may have been misdiagnosed as aseptic failure rather than PJI and subsequently not included in the study. The numbers of patients in each group are small and unequal, although these represent relatively large groups compared with the previous literature concerning oncological PJI.

## 6 Conclusions

Despite empirical antibiotic prophylaxis and empirical management of PJI for oncological EPR being based upon the management of arthroplasty PJI, there are notable differences regarding their host and soft-tissue status. Oncology patients suffered worse host and extremity criteria. Coagulase-negative *Staphylococcus* species were the most common infective organism in both study groups; however, oncology EPR PJI has a significantly higher incidence of MDR infection. Therefore, the authors would recommend broad-spectrum empirical antibiotics pre-emptively when oncological patients undergo revision. The common finding of MDR would support the preferential strategy of a two-stage revision procedure over a single-stage revision procedure which is the practice within our institution. Finally, the rarity of fungal organisms, even in complex oncological cases, would not support the use of empirical antifungal treatments unless confirmed with preoperative sampling.

## Supplement

10.5194/jbji-7-177-2022-supplement The supplement related to this article is available online at: https://doi.org/10.5194/jbji-7-177-2022-supplement.

## Data Availability

All raw data can be provided by the corresponding authors upon request.

## References

[bib1.bib1] Bosco JA, Bookman J, Slover J, Edusei E, Levine B (2015). Principles of antibiotic prophylaxis in total joint arthroplasty: current concepts. J Am Acad Orthoph Sur.

[bib1.bib2] Christensen DD, Moschetti WE, Brown MG, Lucas AP, Jevsevar DS, Fillingham YA, Center DH (2021). Perioperative Antibiotic Prophylaxis: Single and 24-Hour Antibiotic Dosages are Equally Effective at Preventing Periprosthetic Joint Infection in Total Joint Arthroplasty. J Arthroplasty.

[bib1.bib3] Cirstoiu C, Cretu B, Serban B, Panti Z, Nica M (2019). Current review of surgical management options for extremity bone sarcomas. EFORT open reviews.

[bib1.bib4] Dhanoa A, Ajit Singh V, Elbahri H (2015). Deep infections after endoprosthetic replacement operations in orthopedic oncology patients. Surg Infect (Larchmt).

[bib1.bib5] Fehring KA, Abdel MP, Ollivier M, Mabry TM, Hanssen AD (2017). Repeat two-stage exchange arthroplasty for periprosthetic knee infection is dependent on host grade. J Bone Joint Surg Am.

[bib1.bib6] Fogelberg EV, Zitzmann EK, Stinchfield FE (1970). Prophylactic penicillin in orthopaedic surgery. J Bone Joint Surg Am.

[bib1.bib7] Gallo J, Kolár M, Koukalová D, Sauer P, Lovecková Y, Dendis M, Kesselová M, Petrzelová J, Yapletalová J (2006). Bacterial pathogens of periprosthetic infections and diagnostic possibilities. Klin Mikrobiol Infekc Lek.

[bib1.bib8] Gosheger G, Gebert C, Ahrens H, Streitbuerger A, Winkelmann W, Hardes J (2006). Endoprosthetic reconstruction in 250 patients with sarcoma. Clin Orthop Relat R.

[bib1.bib9] Jeys L, Grimer R (2009). The long-term risks of infection and amputation with limb salvage surgery using endoprostheses. Recent Results Cancer Res.

[bib1.bib10] Jeys LM, Grimer RJ, Carter SR, Tillman RM (2005). Periprosthetic infection in patients treated for an orthopaedic oncological condition. J Bone Joint Surg Am.

[bib1.bib11] Kapadia BH, Berg RA, Daley JA, Fritz J, Bhave A, Mont MA (2016). Periprosthetic joint infection. Lancet.

[bib1.bib12] Legout L, Senneville E, Stern R, Yazdanpanah Y, Savage C, Roussel-Delvalez M, Rosele B, Migaud H, Mouton Y (2006). Treatment of bone and joint infections caused by Gram-negative bacilli with a cefepime-fluoroquinolone combination. Clin Microbiol Infect.

[bib1.bib13] Li C, Renz N, Trampuz A (2018). Management of periprosthetic joint infection. Hip Pelvis.

[bib1.bib14] Li C, Renz N, Trampuz A, Ojeda-Thies C (2020). Twenty common errors in the diagnosis and treatment of periprosthetic joint infection. Int Orthop.

[bib1.bib15] McPherson EJ, Woodson C, Holtom P, Roidis N, Shufelt C, Patzakis M (2002). Periprosthetic Total Hip Infection: Outcomes Using a Staging System. Clin Orthop Relat Res.

[bib1.bib16] Morii T, Morioka H, Ueda T, Araki N, Hashimoto N, Kawai A, Mochizuki K, Ichimura S (2013). Deep infection in tumor endoprosthesis around the knee: A multi-institutional study by the Japanese musculoskeletal oncology group. BMC Musculoskelet Disord.

[bib1.bib17] Nickinson RSJ, Board TN, Gambhir AK, Porter ML, Kay PR (2010). The microbiology of the infected knee arthroplasty. Int Orthop.

[bib1.bib18] Pantosti A, Sanchini A, Monaco M (2007). Mechanisms of antibiotic resistance in Staphylococcus aureus. Future Microbiol.

[bib1.bib19] Parvizi J, Tan TL, Goswami K, Higuera C, Della Valle C, Chen AF, Shohat N (2018). The 2018 definition of periprosthetic hip and knee infection: an evidence-based and validated criteria. J Arthroplasty.

[bib1.bib20] Pulido L, Ghanem E, Joshi A, Purtill JJ, Parvizi J (2008). Periprosthetic joint infection: the incidence, timing, and predisposing factors. Clin Orthop Relat R.

[bib1.bib21] Racano A, Pazionis T, Farrokhyar F, Deheshi B, Ghert M (2013). High infection rate outcomes in long-bone tumor surgery 70 with endoprosthetic reconstruction in adults: a systematic review. Clin Orthop Relat R.

[bib1.bib22] Tsai Y, Chang CH, Lin YC, Lee SH, Hsieh PH, Chang Y (2019). Different microbiological profiles between hip and knee prosthetic joint infections. J Orthop Surg.

[bib1.bib23] Tsukayama DT, Estrada R, Gustilo RB (1996). Infection after total hip
arthroplasty: A study of the treatment of one hundred and six infections. J Bone Joint Surg Am.

[bib1.bib24] Wafa H, Grimer RJ (2006). Surgical options and outcomes in bone sarcoma. Expert Rev Anticanc.

[bib1.bib25] Zmistowski B, Alijanipour P (2014). Periprosthetic joint infection of the hip and knee.

