# Supplementary material for: A comparison of the microbiology profile for periprosthetic joint infection of knee arthroplasty and lower-limb endoprostheses in tumour surgery
Source: J Bone Jt Infect. 2022 Aug 8;7(4):177–82. doi: 10.5194/jbji-7-177-2022 (PMC9399934; doi:10.5194/jbji-7-177-2022)
Supplement: The supplement related to this article is available online at: https://doi.org/10.5194/jbji-7-177-2022-supplement. [file jbji-7-177-supplement.pdf]

Supplement of J. Bone Joint Infect., 7, 177–182, 2022
https://doi.org/10.5194/jbji-7-177-2022-supplement
© Author(s) 2022. CC BY 4.0 License.

Supplement of

A comparison of the microbiology profile for periprosthetic joint infection of knee arthroplasty and lower-limb endoprostheses in tumour surgery

Robert A. McCulloch et al.

Correspondence to: Robert A. McCulloch (robert.mcculloch@nhs.net)

The copyright of individual parts of the supplement might differ from the article licence.

Table S1. A chronological summary of cultured organisms during the study period

Count

		PrimaryKnee_Endoprosthesis		
		Primary Knee	Endoprosthesis	Total
Micro_Chart	Alpha haemolytic streptococcus	1	2	3
	Arcano. Haemolyticum	1	0	1
	Beta haemolytic streptococcus	4	7	11
	Coagulase negative staphylococcus	36	23	59
	Corynebacterium sp	2	2	4
	Cutibacterium sp	3	0	3
	Enterobacter sp	2	1	3
	Enterococcus sp	4	2	6
	Escherichia coli	3	2	5
	Finegoldia magna	1	0	1
	Fungal	1	0	1
	Granulicatella adiacens	0	1	1
	Klebsiella sp	0	1	1
	Kocuria kristinae	1	0	1
	Morganella morganii	2	0	2
	MRSA	0	1	1
	Mycobacteria sp	1	0	1
	Negative Culture	6	4	10
	Pseudomonas sp	2	0	2
	Serratia sp	1	1	2
	Staphylococcus aureus	16	7	23
	VRE	0	1	1
Total		87	55	142
